# Copper-mediated DNA damage caused by purpurin, a natural anthraquinone

**DOI:** 10.1186/s41021-022-00245-2

**Published:** 2022-05-09

**Authors:** Hatasu Kobayashi, Yurie Mori, Ryo Iwasa, Yuichiro Hirao, Shinya Kato, Shosuke Kawanishi, Mariko Murata, Shinji Oikawa

**Affiliations:** 1 Department of Environmental and Molecular medicine, Mie University Graduate School of Medicine, Edobashi 2-174, Tsu, Mie 514-8507 Japan; 2 Faculty of Pharmacy, Gifu University of Medical Science, 4-3-3 Nijigaoka, Kani, Gifu 509-0293 Japan; 3 Mie Prefectural College of Nursing, Yumegaoka 1-1-1, Tsu, Mie 514-0116 Japan; 4 Radioisotope Experimental Facility, Advanced Science Research Promotion Center, Mie University, Edobashi 2-174, Tsu, Mie 514-8507 Japan; 5 Faculty of Pharmaceutical Science, Suzuka University of Medical Science, 3500-3, Minamitamagaki, Suzuka, Mie 513-8670 Japan

**Keywords:** Purpurin, Copper, DNA damage, Reactive oxygen species

## Abstract

**Background:**

Purpurin (1,2,4-trihydroxy-9,10-anthraquinone), a natural red anthraquinone pigment, has historically been used as a textile dye. However, purpurin induced urinary bladder tumors in rats, and displayed a mutagenic activity in assay using bacteria and mammalian cells. Many carcinogenic dyes are known to induce bladder cancers via DNA adduct formation, but carcinogenic mechanisms of purpurin remain unknown. In this study, to clarify the mechanism underlying carcinogenicity of purpurin, copper-mediated DNA damage induced by purpurin was examined using ^32^P-labeled DNA fragments of human genes relevant to cancer. Furthermore, we also measured 8-oxo-7,8-dihydro-2′-deoxyguanosine (8-oxodG), an indicator of oxidative DNA damage, in calf thymus DNA.

**Results:**

Purpurin plus Cu(II) cleaved ^32^P-labeled DNA fragments only under piperidine treatment, indicating that purpurin caused base modification, but not breakage of the DNA backbone. In the absence of Cu(II), purpurin did not induce DNA cleavage even with piperidine treatment. Purpurin plus Cu(II) caused piperidine-labile sites predominantly at G and some T residues. Bathocuproine, a Cu(I) chelator, completely prevented the occurrence of piperidine-labile sites, indicating a critical role of Cu(I) in piperidine-labile sites induced by purpurin plus Cu(II). On the other hand, methional, a scavenger of a variety of reactive oxygen species (ROS) and catalase showed limited inhibitory effects on the induction of piperidine-labile sites, suggesting that ROS could not be major mediators of the purpurin-induced DNA damage. Considering reported DNA adduct formation by quinone metabolites of several carcinogenic agents, quinone form of purpurin, which is possibly generated via purpurin autoxidation accompanied by Cu(I)/Cu(II) redox cycle, might lead to DNA adducts and piperidine-labile sites. In addition, we measured contents of 8-oxodG. Purpurin moderately but significantly increased 8-oxodG in calf thymus DNA in the presence of Cu(II). The 8-oxodG formation was inhibited by catalase, methional and bathocuproine, suggesting that Cu(I)-hydroperoxide, which was generated via Cu(I) and H_2_O_2_, caused oxidative DNA base damage.

**Conclusions:**

We demonstrated that purpurin induces DNA base damage possibly mediated by Cu(I)/Cu(II) redox cycle both with and without ROS generation, which are likely to play an important role in its carcinogenicity.

**Supplementary Information:**

The online version contains supplementary material available at 10.1186/s41021-022-00245-2.

## Introduction

Anthraquinones are a class of organic compounds abundant in the universe of natural substances and are found in various plant parts such as roots, rhizomes, fruits, and flowers [1]. Purpurin (1,2,4-trihydroxy-9,10-anthraquinone) is a natural red anthraquinone pigment mostly isolated from madder root (*Rubia tinctorum*) [2]. Historically, purpurin has been used not only as a textile dye but also as an ingredient of herbal medicine and a food coloring agent. Purpurin has been reported to show beneficial biological activities such as antioxidant, antimutagenic, antimicrobial, neuroprotective, antiadipogenic, and anticancer activities [2].

On the other hand, purpurin was reported to induce marked hyperplasia of pelvic epithelium and urinary bladder tumors (papilloma and carcinoma) in rats [3]. Purpurin had also a mutagenic activity in Salmonella without mammalian microsomal activation [4, 5] and was active in various mutagenesis assay systems using mammalian cells including V79-HGPRT assay, DNA-repair assay in primary rat hepatocytes, and transformation of C3H/M2 mouse fibroblasts [5]. Working exposed to dyes has been identified as one of the major risk factors for occupational bladder cancer [6, 7]. Many carcinogenic dyes induce carcinogenesis via DNA adduct formation, but carcinogenic mechanisms of purpurin remain unknown.

In this study, to clarify the mechanism underlying the carcinogenicity of purpurin, we investigated purpurin-induced DNA damage in the presence of copper ions. We analyzed DNA damage using ^32^P-5′-end-labeled DNA fragments obtained from the c-Ha-*ras*-1 protooncogene and the *p16* tumor suppressor gene. We also measured the contents of 8-oxo-7,8-dihydro-2′-deoxyguanosine (8-oxodG), a relevant indicator of oxidative DNA damage, in calf thymus DNA using a high performance liquid chromatograph equipped with an electrochemical detector (HPLC-ECD).

## Materials and methods

### Materials

*Eco*RI, *Ava*I, *Pst*I and T_4_ polynucleotide kinase were purchased from New England Biolabs Ltd. (Ipswich, MA, USA). *Bss*HII was purchased from Takara Bio Inc. (Shiga, Japan). [γ-^32^P] ATP (222 TBq/mmol) was purchased from Perkin Elmer, Inc. (Waltham, MA, USA). Purpurin, catalase (30,000 units/mg from bovine liver) and 3-(methylthio) propionaldehyde (methional) were purchased from Sigma-Aldrich Co., LLC. (St Louis, MO, USA). Copper chloride dihydrate (CuCl_2_·2H_2_O), ethanol, mannitol and sodium formate were purchased from Nacalai Tesque (Kyoto, Japan). Diethylenetriamine-*N,N,N′,N″,N″*-pentaacetic acid (DTPA) and bathocuproine disulfonic acid were purchased from Dojindo Laboratories (Kumamoto, Japan). Nuclease P_1_ (500 units/vial) and piperidine were purchased from FUJIFILM Wako Pure Chemical Co., Ltd. (Osaka, Japan). Calf intestinal phosphatase (500 units/vial) was purchased from Roche Diagnostics GmbH (Mannheim, Germany).

### Preparation of ^32^P-5′-end-labeled DNA fragments

DNA fragments were obtained from the human *p16* tumor suppressor gene [8] and the c-Ha-*ras*-1 protooncogene [9]. A fragment containing exon 2 of the human *p16* tumor suppressor gene was obtained as described previously [10]. The 5′ end-labeled 460-base pair fragment (*Eco*RI* 9481-*Eco*RI* 9940) containing exon 2 was also further digested with *Bss*HII to obtain the singly labeled 309-base pair fragment (*Eco*RI* 9481-*Bss*HII 9789) and the 147-base pair fragment (*Bss*HII 9794-*Eco*RI* 9940). DNA fragments were also prepared from plasmid pbcNI, which carries a 6.6-kb *Bam*HI chromosomal DNA segment containing the human c-Ha-*ras*-1 protooncogene. The singly labeled 98-base pair fragment (*Ava*I* 2247-*Pst*I 2344) and 337-base pair fragment (*Pst*I 2345-*Ava*I* 2681) were obtained as previously described [11]. For reference, nucleotide numbering starts with the *Bam*HI site [9]. An asterisk indicates ^32^P-labeling.

### Detection of damage to isolated ^32^P-labeled DNA fragments

Reaction mixtures containing ^32^P-labeled DNA fragment, 20 μM/base calf thymus DNA, purpurin and 20 μM CuCl_2_ in 200 μl of sodium phosphate buffer (pH 7.8) containing 5 μM DTPA were incubated for 1 h at 37 °C under light shielding. To examine the effects of reactive oxygen species (ROS) scavengers and bathocuproine, these reagents were added before the addition of purpurin. The DNA fragments were heated in 1 M piperidine for 20 min at 90 °C, followed by electrophoresis on an 8% polyacrylamide/8 M urea gel as previously described [11]. An autoradiogram was obtained by exposing an X-ray film (FUJIFILM Corp., Tokyo, Japan) to the gel. Band intensity was quantified using ImageJ software (National Institutes of Health).

The preferred cleavage sites were determined by directly comparing the positions of the oligonucleotides with those produced by the chemical reactions of the Maxam-Gilbert procedure [12], using a DNA-sequencing system (LKB 2010 Macrophor, LKB Pharmacia Biotechnology Inc., Uppsala, Sweden). The autoradiogram was obtained by exposing an imaging plate (BAS-MS2040, FUJIFILM Corp.) to the gel. The relative amounts of oligonucleotides from the treated DNA fragments were measured using a laser scanner (Typhoon FLA-9500, GE Healthcare, Buckinghamshire, England) and analyzed with ImageQuant TL software (GE Healthcare).

### Analysis of 8-oxodG formation in calf thymus DNA

Measurement of 8-oxodG in calf thymus DNA was performed as described previously [13]. The reaction mixtures, containing 100 μM/base calf thymus DNA, purpurin and 20 μM CuCl_2_ in 400 μl of 4 mM sodium phosphate buffer (pH 7.8) containing 5 μM DTPA, were incubated for 1 h at 37 °C under light shielding. To examine the effects of ROS scavengers and bathocuproine, these reagents were added before the addition of purpurin. After ethanol precipitation, the DNA was digested into nucleosides with nuclease P_1_ and calf intestine phosphatase. The amount of 8-oxodG was measured using an HPLC-ECD as described previously [14].

### Statistical analysis

Results were presented as means ± standard deviation (SD). Differences were analyzed by student t-test. *P* values less than 0.05 were considered to be statistically significant.

## Results

### Damage of ^32^P-labeled DNA fragments by purpurin in the presence of Cu(II)

**Fig. 1:**
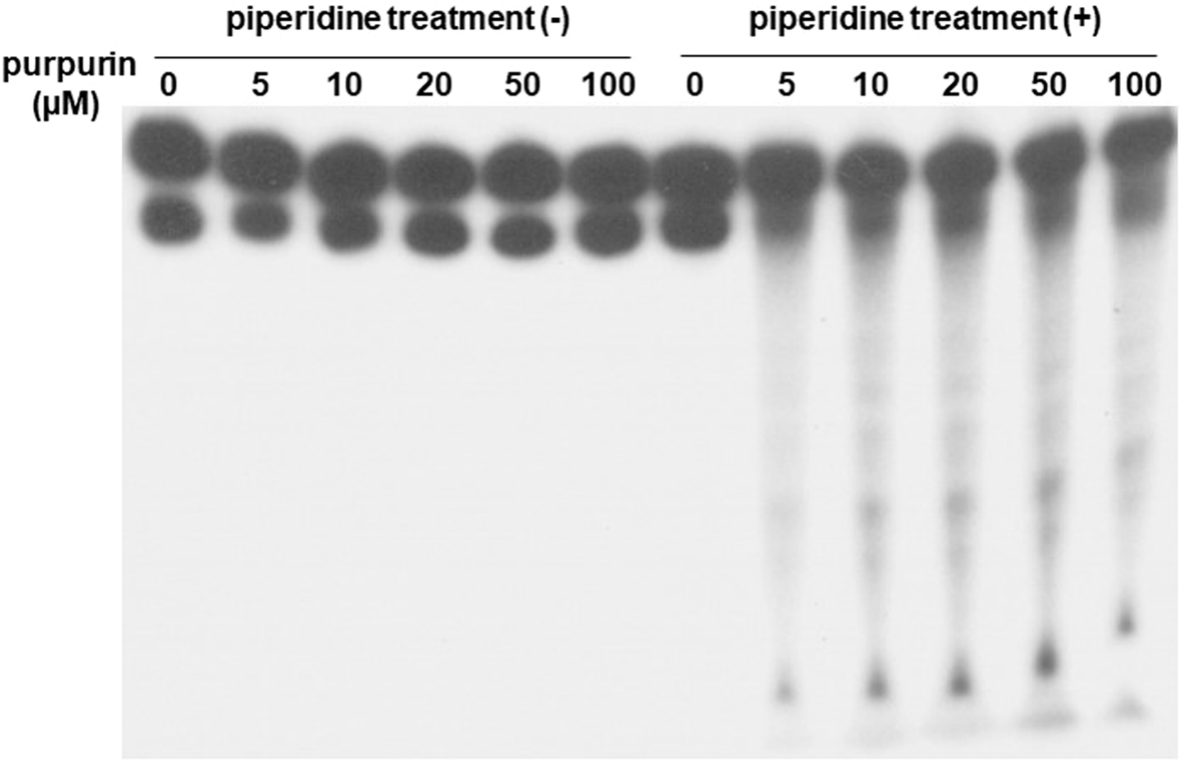
Autoradiogram of ^32^P-5′-end-labeled DNA fragments incubated with purpurin in the presence of Cu(II). Reaction mixtures contained the ^32^P-5′-end-labeled 147-bp fragment, 20 μM/base calf thymus DNA, the indicated concentrations of purpurin and 20 μM CuCl_2_ in 200 μl of 4 mM sodium phosphate buffer (pH 7.8) containing 5 μM DTPA. After incubation at 37 °C for 1 h under light shielding, the DNA fragments were treated with or without hot piperidine and electrophoresed on a polyacrylamide gel

Figure 1 shows the autoradiogram of DNA fragments incubated with purpurin plus Cu(II) and followed by treatment with or without piperidine. Oligonucleotides were detected on the autoradiogram as a result of the DNA cleavage. In the presence of Cu(II), purpurin caused DNA cleavage with piperidine treatment, although no or little DNA cleavage was observed without piperidine treatment (Fig. 1 and Fig. S1A). Meanwhile, in the absence of Cu(II), purpurin did not induce DNA cleavage even with piperidine treatment (data not shown). DNA cleavage induced by purpurin plus Cu(II) was detected at 5 μM of purpurin, and the degree of the DNA cleavage was increased at 10, 20, 50 and 100 μM. As piperidine cleaves DNA at sugars with modified bases, it is reasonable to conclude that purpurin caused base modification but not breakage of deoxyribose phosphate backbone in the presence of Cu(II).

### Effects of scavengers and bathocuproine on DNA damage induced by purpurin in the presence of Cu(II)

**Fig. 2:**
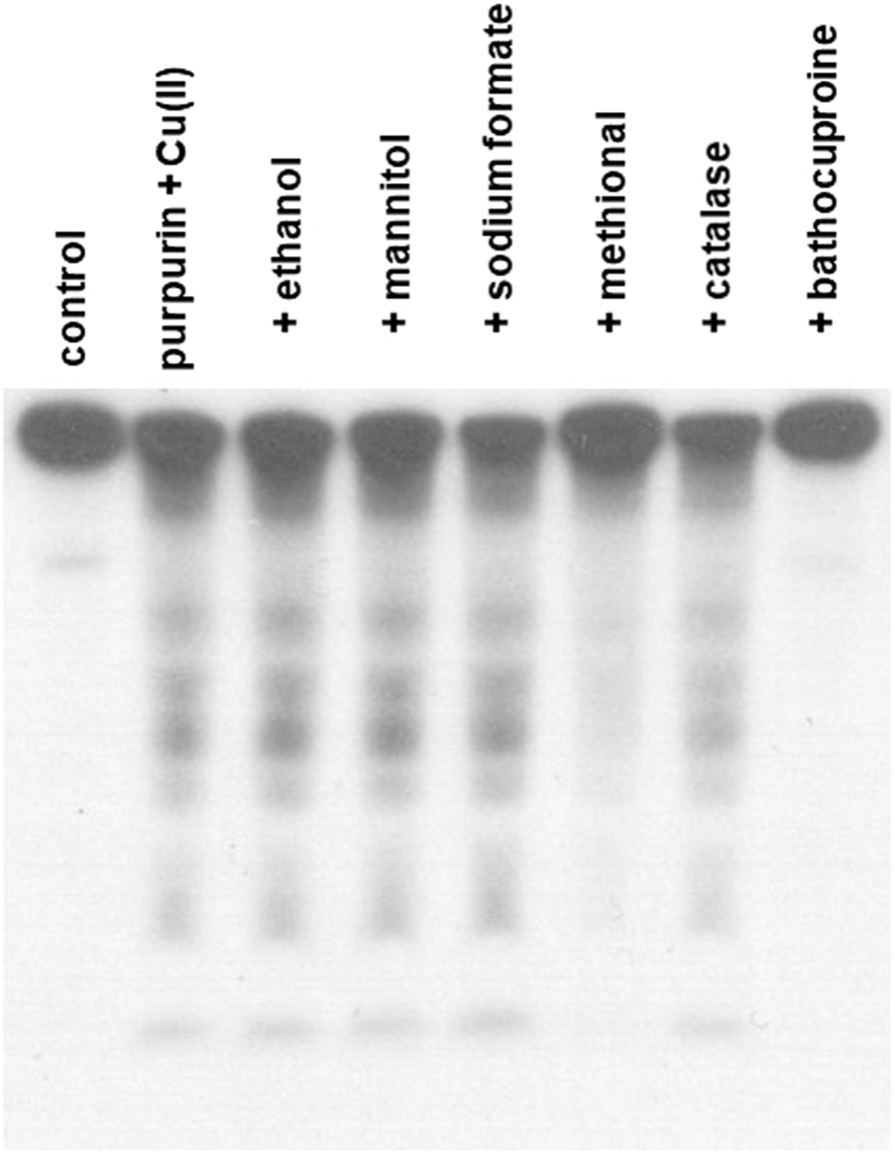
Effects of ROS scavengers and bathocuproine on purpurin-induced DNA damage in ^32^P-5′-end-labeled DNA fragments. Reaction mixtures contained the ^32^P-5′-end-labeled 147-bp fragment, 20 μM/base calf thymus DNA, 50 μM purpurin, each scavenger or bathocuproine and 20 μM CuCl_2_ in 200 μl of 4 mM sodium phosphate buffer (pH 7.8) containing 5 μM DTPA. After incubation at 37 °C for 1 h under light shielding, the DNA fragments were treated with piperidine and electrophoresed on a polyacrylamide gel. The concentrations of each scavenger and bathocuproine were as follows: 0.2 M ethanol, 0.1 M mannitol, 0.1 M sodium formate, 0.6 M methional, 50 U catalase, 50 μM bathocuproine

Effects of the scavengers and bathocuproine on the damage of ^32^P-labeled DNA fragments by purpurin plus Cu(II) were examined. Bathocuproine, a Cu(I)-specific chelator [15], prevented purpurin-induced DNA damage in ^32^P-labeled DNA fragments in the presence of Cu(II) (Fig. 2 and Fig. S1B). Furthermore, methional, a scavenger of a variety of ROS [16], partially inhibited the DNA damage. Catalase also showed a slight inhibitory effect on the DNA damage. On the other hand, the DNA damage was not prevented by typical free hydroxyl radical (•OH) scavengers such as ethanol, mannitol and sodium formate. These results suggest that ROS could not be major mediators of the purpurin-induced DNA damage.

### Site specificity of DNA damage by purpurin in the presence of Cu(II)

**Fig. 3:**
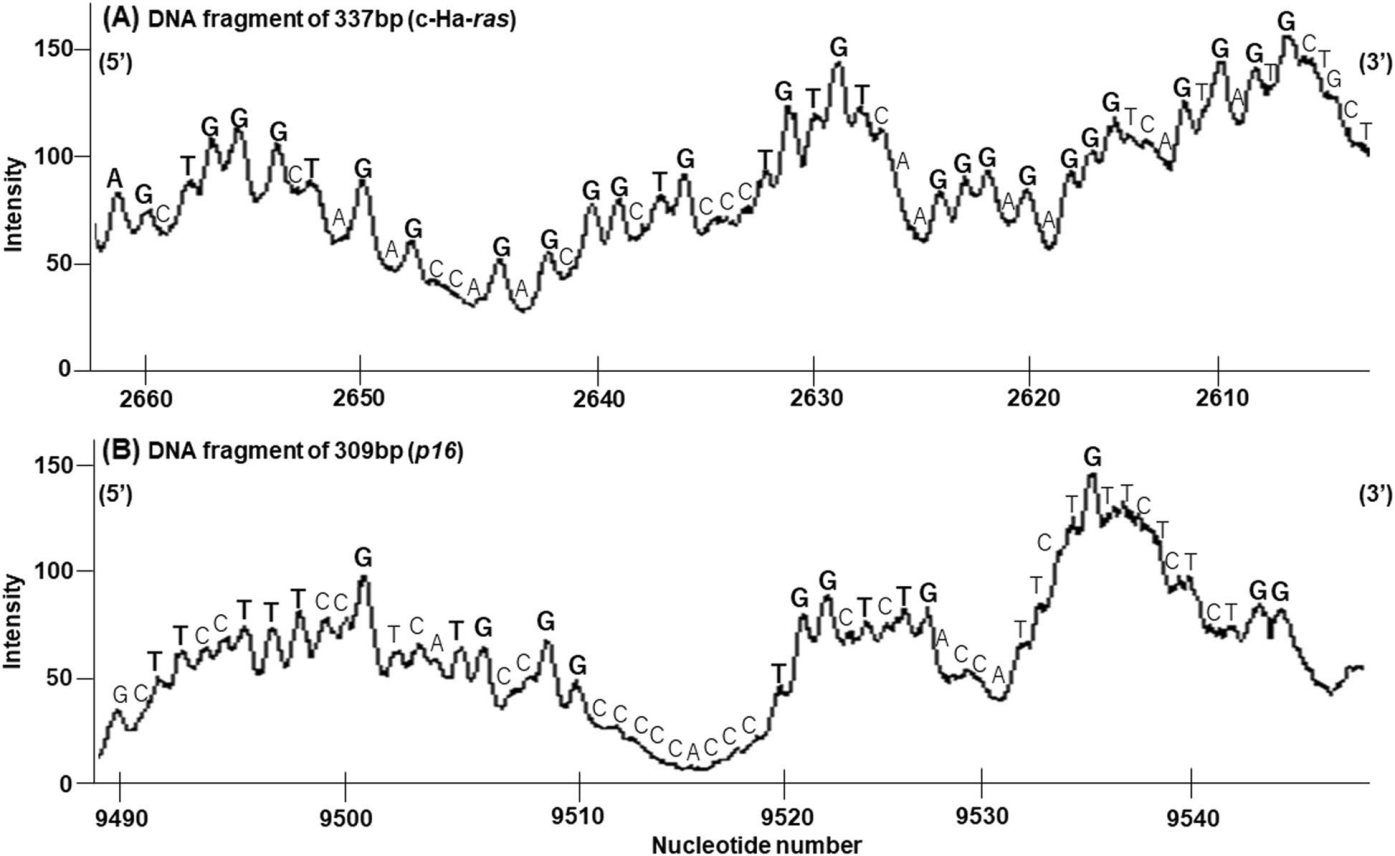
Site specificity of purpurin-induced DNA damage in ^32^P-5′-end-labeled DNA fragments. Reaction mixtures contained the ^32^P-5′-end-labeled (**A**) the 337-bp fragment or (**B**) the 309-bp fragment, 20 μM/base calf thymus DNA, 50 μM purpurin and 20 μM CuCl_2_ in 200 μl of 10 mM sodium phosphate buffer (pH 7.8) containing 5 μM DTPA. After incubation at 37 °C for 1 h under light shielding, the DNA fragments were treated with piperidine and electrophoresed on a polyacrylamide gel. DNA bases in bold represent preferentially cleaved bases

The patterns of DNA damage induced by purpurin in the presence of Cu(II) were determined by DNA sequencing using the Maxam-Gilbert procedure [12]. The relative intensity of DNA damage obtained by scanning autoradiogram with a laser scanner is shown in Fig. 3. Purpurin cleaved DNA frequently at guanine (G) and at some thymine (T) residues in DNA fragments obtained from the human c-Ha-*ras*-1 protooncogene with piperidine treatment (Fig. 3A). In DNA fragments obtained from the *p16* tumor suppressor genes, DNA cleavages at T as well as G residues were induced by purpurin under piperidine treatment (Fig. 3B). Thus, purpurin caused piperidine-labile sites predominantly at G and some T residues.

### Formation of 8-oxodG in calf thymus DNA by purpurin in the presence of Cu(II)

**Fig. 4:**
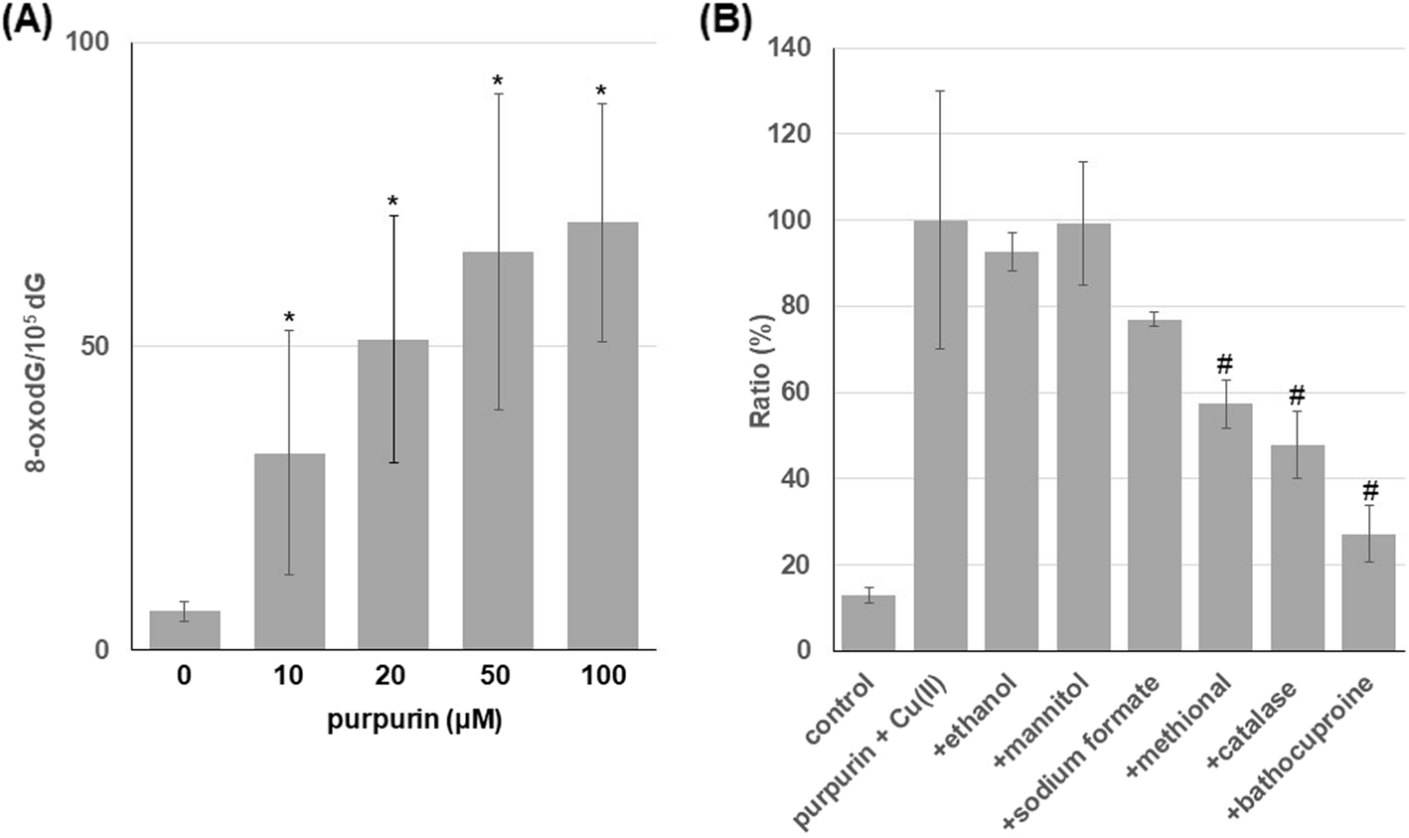
Formation of 8-oxodG in calf thymus DNA by purpurin in the presence of Cu(II). (**A**) Reaction mixtures contained 100 μM/base calf thymus DNA, the indicated concentrations of purpurin and 20 μM CuCl_2_ in 400 μl of 4 mM sodium phosphate buffer (pH 7.8) containing 5 μM DTPA. (**B**) Reaction mixtures contained 100 μM/base calf thymus DNA, 100 μM purpurin, 20 μM CuCl_2_ and each scavenger or bathocuproine in 400 μl of 4 mM sodium phosphate buffer (pH 7.8) containing 5 μM DTPA. The concentrations of each scavenger and bathocuproine were as follows: 0.2 M ethanol, 0.1 M mannitol, 0.1 M sodium formate, 0.6 M methional, 50 U catalase, 50 μM bathocuproine. Reaction mixtures were incubated at 37 °C for 1 h under light shielding. After ethanol precipitation, the DNA was digested to nucleosides with nuclease P_1_ and calf intestine phosphatase, then analyzed with an HPLC-ECD system. * *p* < 0.05 vs 0 μM. # *p* < 0.05 vs purpurin plus Cu(II). Significance was analyzed using Student t-test

To investigate oxidative DNA damage, we measured the contents of 8-oxodG, an indicator of oxidative base damage, in calf thymus DNA treated with purpurin in the presence of Cu(II). The formation of 8-oxodG was significantly increased by purpurin treatment in a concentration-dependent manner (Fig. 4A). Figure 4B shows the effects of ROS scavengers and bathocuproine on the Cu(II)-mediated 8-oxodG formation induced by purpurin. Catalase and methional significantly inhibited the 8-oxodG formation, while typical •OH scavengers such as ethanol, mannitol and sodium formate did not. Bathocuproine showed an inhibitory effect on the 8-oxodG formation. These results suggest the involvement of H_2_O_2_, ROS other than •OH and Cu(I) in the 8-oxodG formation caused by purpurin plus Cu(II).

## Discussion

**Fig. 5:**
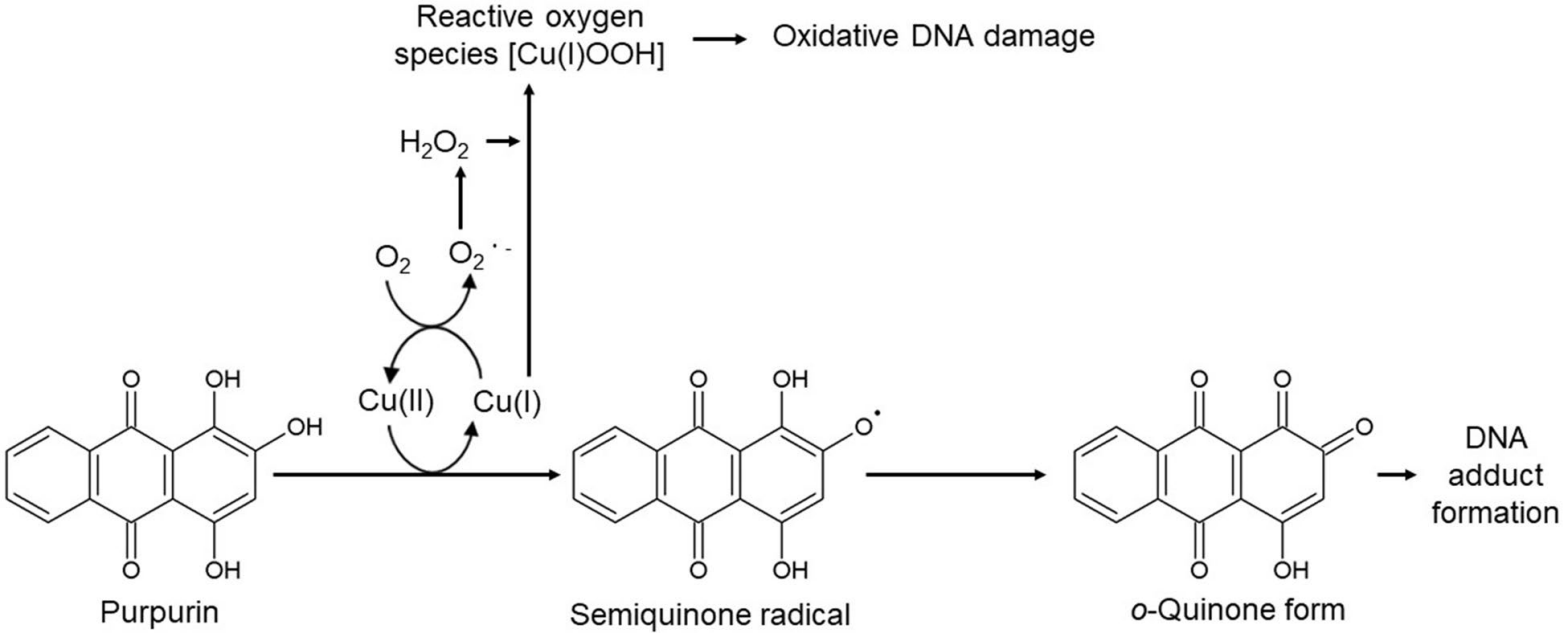
A possible mechanism of DNA base damage induced by purpurin and Cu(II)

The present study demonstrated that purpurin caused copper-mediated DNA damage in ^32^P-labeled DNA fragments. Cleaved oligonucleotides were observed only under piperidine treatment, indicating that purpurin caused base modification, but not breakage of the deoxyribose-phosphate backbone, in the presence of Cu(II). Bathocuproine completely prevented the piperidine-labile sites induced by purpurin plus Cu(II), while methional and catalase also displayed limited inhibitory effects on the DNA damage (Fig. 2). Site specificity of the purpurin-induced piperidine-labile sites (predominantly at G) is inconsistent with our previous studies indicating that many ROS-generating agents plus Cu(II) frequently cleaved DNA at cytosine (C) and T under piperidine treatment [17–19]. These results suggest that mechanisms other than ROS generation could mainly contribute to purpurin-induced DNA damage. Some DNA adducts formed in DNA base has been reported to lead to DNA strand break under piperidine treatment [20–22]. Many quinones are reported to form DNA adduct through covalently binding to nucleobases of DNA [23]. Notably, *o*-quinone metabolites of carcinogens such as estrogen and bisphenol A contribute to their carcinogenic mechanism via DNA adduct formation [24, 25]. Thus, we considered that the *o*-quinone form of purpurin, which is generated via purpurin autoxidation accompanied by Cu(I)/Cu(II) redox cycle, could lead to DNA adducts and piperidine-labile sites (Fig. 5). Although a recent study has reported that purpurin can react with Cu(II) generating a metal complex (2:1) under certain conditions [26], the complex was not detected by ultraviolet-visible spectroscopy under our conditions (data not shown).

Interestingly, the present study indicated that purpurin plus Cu(II) also induced oxidative damage. We examined the ability of purpurin to induce 8-oxodG formation. 8-OxodG causes DNA misreplication resulting in mutation or cancer [27, 28] and is one of the oxidative DNA products generated by the reaction with ROS [29]. Purpurin modestly but significantly increased formation of 8-oxodG in calf thymus DNA in the presence of Cu(II). To clarify the mechanism of purpurin-induced 8-oxodG formation, we investigated effects of ROS scavengers and bathocuproine. Catalase and bathocuproine exhibited inhibitory effects on the increase of 8-oxodG, although not complete. Methional also showed similar results, while •OH scavengers did not inhibit the 8-oxodG formation. These results suggest that Cu(I)/Cu(II) redox cycle and concomitant H_2_O_2_ production possibly via autoxidation of purpurin lead to generate Cu(I)-hydroperoxide, which induces oxidative DNA base damage including 8-oxodG formation (Fig. 5).

A higher level of serum copper was observed in bladder cancer patients than in healthy controls [30]. A recent case-controlled clinical study reported that plasma level of copper was significantly and positively associated with bladder cancer development, suggesting that the increase in plasma copper may be one of the risk factors for bladder cancer [31]. Many studies have reported that copper plays a role in promoting carcinogenesis though inducing oxidative stress and resultant DNA damage [32–34]. Copper ions have been known to be particularly adept at helping other agents to damage DNA and to enhance mutagenic activities via production of ROS [35, 36]. Our previous studies also demonstrated that copper mediated the various carcinogens-induced ROS production and oxidative DNA damage [19, 37, 38]. However, the present study suggests that purpurin plus Cu(II) leads to DNA damage through not only ROS generation but also possible DNA adduct formation mediated by Cu(I)/Cu(II) redox cycle (Fig. 5). Therefore, both oxidative DNA damage and DNA adduct could be involved in the carcinogenic mechanism of purpurin.

Several previous studies reported that purpurin is also capable of binding DNA based on voltammetric, electrochemical and spectroscopic analysis [39–41]. These findings suggest that purpurin, copper and DNA might coexist and interact with each other and lead to DNA damage via ROS generation and DNA adduct formation. Future research should focus on the association between copper and purpurin for understanding of mechanism of purpurin carcinogenicity.

## Conclusions

We demonstrated that purpurin plus Cu(II) induces Cu(I)/Cu(II) redox cycle-mediated DNA base damage, possibly through both ROS-independent and -dependent mechanisms, which are likely to contribute to its carcinogenicity.

## Supplementary Information

**Additional file 1.**

## Data Availability

Data sharing is not applicable to this article as no datasets were generated or analyzed during the current study.

## Abbreviations

8-oxodG: 8-oxo-7,8-dihydro-2′-deoxyguanosine
DTPA: Diethylenetriamine-N,N,N′,N″,N″-pentaacetic acid
HPLC-ECD: A high performance liquid chromatograph equipped with an electrochemical detector
ROS: Reactive oxygen species

## References

[1] Diaz-Muñoz G, Miranda IL, Sartori SK, de Rezende DC, MAN D (2018). Anthraquinones: An Overview. Stud Nat Prod Chem.

[2] Singh J, Hussain Y, Luqman S, Meena A. Purpurin: a natural anthraquinone with multifaceted pharmacological activities. Phytother Res. 2021;35(5):2418–28. 10.1002/ptr.6965. 10.1002/ptr.6965 33254282

[3] Mori H, Ohnishi M, Kawamori T, Sugie S, Tanaka T, Ino N (1996). Toxicity and tumorigenicity of purpurin, a natural hydroxanthraquinone in rats: induction of bladder neoplasms. Cancer Lett.

[4] Brown JP, Brown RJ (1976). Mutagenesis by 9,10-anthraquinone derivatives and related compounds in Salmonella typhimurium. Mutat Res.

[5] Westendorf J, Marquardt H, Poginsky B, Dominiak M, Schmidt J, Marquardt H (1990). Genotoxicity of naturally occurring hydroxyanthraquinones. Mutat Res.

[6] Cumberbatch MG, Windsor-Shellard B, Catto JW (2017). The contemporary landscape of occupational bladder cancer within the United Kingdom: a meta-analysis of risks over the last 80 years. BJU Int.

[7] Cumberbatch MG, Cox A, Teare D, Catto JW (2015). Contemporary occupational carcinogen exposure and bladder Cancer: a systematic review and Meta-analysis. JAMA Oncol.

[8] Serrano M, Hannon GJ, Beach D (1993). A new regulatory motif in cell-cycle control causing specific inhibition of cyclin D/CDK4. Nature.

[9] Capon DJ, Chen EY, Levinson AD, Seeburg PH, Goeddel DV (1983). Complete nucleotide sequences of the T24 human bladder carcinoma oncogene and its normal homologue. Nature.

[10] Bruner SD, Norman DP, Verdine GL (2000). Structural basis for recognition and repair of the endogenous mutagen 8-oxoguanine in DNA. Nature.

[11] Kawanishi S, Yamamoto K (1991). Mechanism of site-specific DNA damage induced by methylhydrazines in the presence of copper (II) or manganese (III). Biochemistry.

[12] Maxam AM, Gilbert W (1980). Sequencing end-labeled DNA with base-specific chemical cleavages. Methods Enzymol.

[13] Kasai H, Crain PF, Kuchino Y, Nishimura S, Ootsuyama A, Tanooka H (1986). Formation of 8-hydroxyguanine moiety in cellular DNA by agents producing oxygen radicals and evidence for its repair. Carcinogenesis.

[14] Ito K, Inoue S, Yamamoto K, Kawanishi S (1993). 8-Hydroxydeoxyguanosine formation at the 5′ site of 5′-GG-3′ sequences in double-stranded DNA by UV radiation with riboflavin. J Biol Chem.

[15] Blair D, Diehl H (1961). Bathophenanthrolinedisulphonic acid and bathocuproinedisulphonic acid, water soluble reagents for iron and copper. Talanta.

[16] Pryor WA, Tang RH (1978). Ethylene formation from methional. Biochem Biophys Res Commun.

[17] Kobayashi H, Murata M, Kawanishi S, Oikawa S (2020). Polyphenols with anti-amyloid beta aggregation show potential risk of toxicity via pro-oxidant properties. Int J Mol Sci.

[18] Oikawa S (2008). Mechanism of oxidative DNA damage induced by environmental carcinogens and antioxidants. Genes Environ.

[19] Murata M, Kawanishi S (2011). Mechanisms of oxidative DNA damage induced by carcinogenic arylamines. Front Biosci (Landmark edition).

[20] Yoon JH, Lee CS (2000). Mapping of altromycin B-DNA adduct at nucleotide resolution in the human genomic DNA by ligation-mediated PCR. Mol Cells.

[21] Muller JG, Kayser LA, Paikoff SJ, Duarte V, Tang N, Perez RJ (1999). Formation of DNA adducts using nickel (II) complexes of redox-active ligands: a comparison of salen and peptide complexes. Coord Chem Rev.

[22] Mattes WB, Lee CS, Laval J, O'Connor TR (1996). Excision of DNA adducts of nitrogen mustards by bacterial and mammalian 3-methyladenine-DNA glycosylases. Carcinogenesis.

[23] Xiong Y, Kaw HY, Zhu L, Wang W. Genotoxicity of quinone: an insight on DNA adducts and its LC-MS-based detection. Crit Rev Environ Sci Technol. 2021. 10.1080/10643389.2021.2001276.

[24] Zhao H, Wei J, Xiang L, Cai Z (2018). Mass spectrometry investigation of DNA adduct formation from bisphenol a quinone metabolite and MCF-7 cell DNA. Talanta.

[25] Cavalieri EL, Rogan EG, Zahid M (2017). Critical depurinating DNA adducts: estrogen adducts in the etiology and prevention of cancer and dopamine adducts in the etiology and prevention of Parkinson's disease. Int J Cancer.

[26] Yuan H, Cheng B, Lei J, Jiang L, Han Z (2021). Promoting photocatalytic CO2 reduction with a molecular copper purpurin chromophore. Nat Commun.

[27] Cheng KC, Cahill DS, Kasai H, Nishimura S, Loeb LA (1992). 8-Hydroxyguanine, an abundant form of oxidative DNA damage, causes GT and AC substitutions. J Biol Chem.

[28] Shibutani S, Takeshita M, Grollman AP (1991). Insertion of specific bases during DNA synthesis past the oxidation-damaged base 8-oxodG. Nature.

[29] Kasai H, Chung MH, Jones DS, Inoue H, Ishikawa H, Kamiya H (1991). 8-Hydroxyguanine, a DNA adduct formed by oxygen radicals: its implication on oxygen radical-involved mutagenesis/carcinogenesis. J Toxicol Sci.

[30] Mao S, Huang S (2013). Zinc and copper levels in bladder cancer: a systematic review and meta-analysis. Biol Trace Elem Res.

[31] Mortada WI, Awadalla A, Khater S, Ahmed A, Hamam ET, El-Zayat M (2020). Copper and zinc levels in plasma and cancerous tissues and their relation with expression of VEGF and HIF-1 in the pathogenesis of muscle invasive urothelial bladder cancer: a case-controlled clinical study. Environ Sci Pollut Res Int.

[32] Theophanides T, Anastassopoulou J (2002). Copper and carcinogenesis. Crit Rev Oncol Hematol.

[33] Maung MT, Carlson A, Olea-Flores M, Elkhadragy L, Schachtschneider KM, Navarro-Tito N (2021). The molecular and cellular basis of copper dysregulation and its relationship with human pathologies. FASEB J.

[34] Kawanishi S, Hiraku Y, Murata M, Oikawa S (2002). The role of metals in site-specific DNA damage with reference to carcinogenesis. Free Radic Biol Med.

[35] Linder MC (2012). The relationship of copper to DNA damage and damage prevention in humans. Mutat Res.

[36] Fujii N, Yano S, Takeshita K (2016). Selective enhancing effect of metal ions on mutagenicity. Genes Environ.

[37] Ohnishi S, Hiraku Y, Hasegawa K, Hirakawa K, Oikawa S, Murata M (2018). Mechanism of oxidative DNA damage induced by metabolites of carcinogenic naphthalene. Mutat Res Genet Toxicol Environ Mutagene.

[38] Murata M, Tezuka T, Ohnishi S, Takamura-Enya T, Hisamatsu Y, Kawanishi S (2006). Carcinogenic 3-nitrobenzanthrone induces oxidative damage to isolated and cellular DNA. Free Radic Biol Med.

[39] Ghosh P, Devi GP, Priya R, Amrita A, Sivaramakrishna A, Babu S (2013). Spectroscopic and in silico evaluation of interaction of DNA with six anthraquinone derivatives. Appl Biochem Biotechnol.

[40] Das P, Guina PS, Mandal PC, Paul M, Paul S, Das S (2011). Cyclic voltammetric studies of 1,2,4-trihydroxy-9,10-anthraquinone, its interaction with calf thymus DNA and anti-leukemic activity on MOLT-4 cell lines: a comparison with anthracycline anticancer drugs. J Phys Org Chem.

[41] Wang QX, Gao F, Yuan XL, Li WQ, Liu AF, Jiao K (2010). Electrochemical studies on the binding of a carcinogenic anthraquinone dye, Purpurin (CI 58 205) with DNA. Dyes Pigments.

