# Supplementary figures and images for: Copper-mediated DNA damage caused by purpurin, a natural anthraquinone
Source: Genes Environ. 2022 May 9;44:15. doi: 10.1186/s41021-022-00245-2 (PMC9082958; doi:10.1186/s41021-022-00245-2)

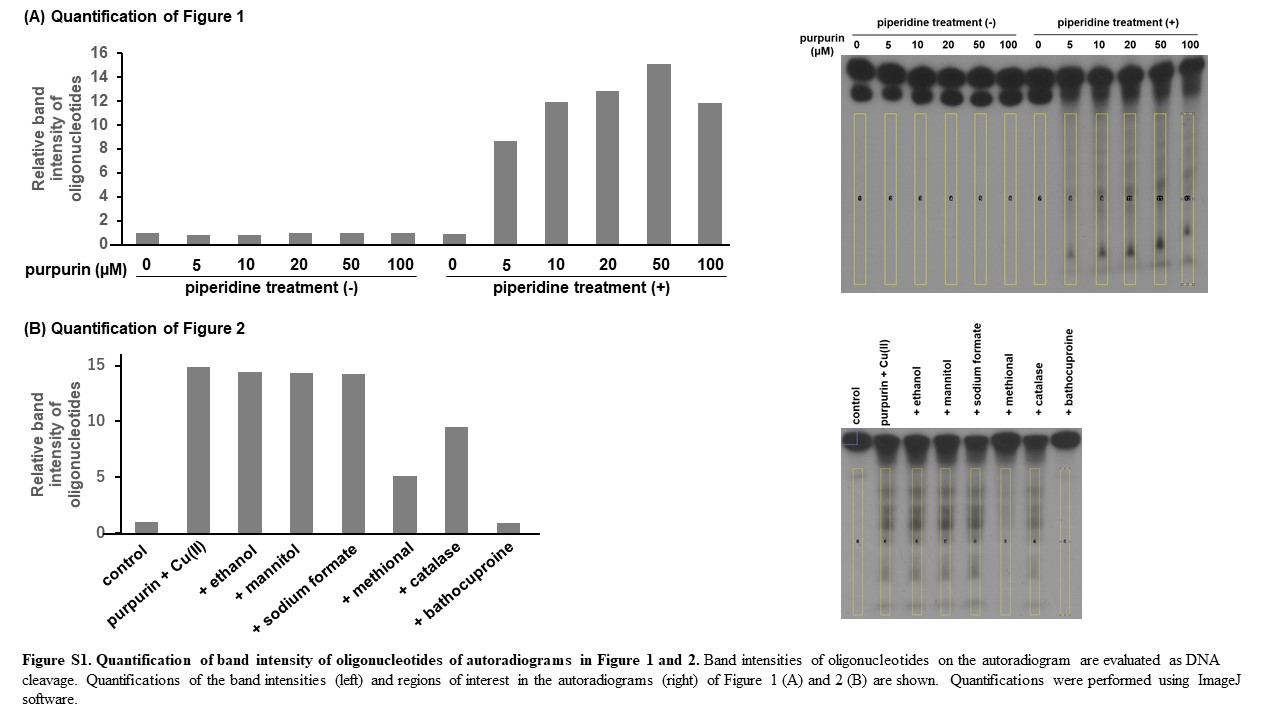

Supplement: Supplementary file 1 — Additional file 1. [file 41021_2022_245_MOESM1_ESM.jpg]
